# Understanding the associations between personality traits and mental health in people with epilepsy and healthy controls

**DOI:** 10.3389/fpsyg.2023.1134188

**Published:** 2023-06-09

**Authors:** Weixi Kang

**Affiliations:** Imperial College London, London, United Kingdom

**Keywords:** mental health, personality, big five, epilepsy, GHQ-12

## Abstract

**Objectives:**

The aim of the current study was to test how epilepsy could moderate the associations between Big Five personality traits and mental health.

**Methods:**

This cross-sectional study analyzed data from Understanding Society: UK Household Longitudinal (UKHLS), which relies on a complex multi-stage stratified sampling design. Personality traits were measured by the Big Five inventory whereas mental health where measured by the GHQ-12. A hierarchical regression and two multiple regressions were performed on 334 people with epilepsy with a mean age of 45.14 ± 15.88 years old (41.32% males) and 26,484 healthy controls (42.5% males) with a mean age of 48.71 ± 17.04 years old.

**Results:**

Neuroticism was positively related to worse mental health in both people with epilepsy and healthy controls with a stronger relationship in people with epilepsy, but Conscientiousness was negatively related to worse mental health in both people with epilepsy and healthy controls. Moreover, Openness and Extraversion were negatively related to worse mental health in healthy controls but not in people with epilepsy.

**Conclusion:**

Personality traits are closely related to mental health in both people with epilepsy and healthy controls. Clinicians should use findings from this study to detect people with epilepsy who may be at high risk of poor mental health based on their personality traits.

## 1. Introduction

Epilepsy is a severe neurological condition that affects approximately 50 million people worldwide. In the United Kingdom alone, there are an estimated 600,000 individuals living with epilepsy (Epilepsy Action, 2018). Furthermore, comorbidities with mental health disorders are highly prevalent among people with epilepsy (Tellez-Zenteno et al., 2007; Fiest et al., 2013; Scott et al., 2017), with approximately 20% of patients experiencing symptoms of depression or anxiety. These mental health challenges can have detrimental effects, including suboptimal adherence to medication regimens, decreased quality of life, lower educational attainment, heightened risks of unemployment, and increased likelihood of suicide (Scott et al., 2017).

Thus, it is important to understand the risk factors for mental health problems, as this knowledge can contribute to the prevention and treatment of comorbid mental health conditions (van der Wal et al., 2018). One such factor is personality, which can be assessed using the Big Five traits, namely Neuroticism, Openness, Agreeableness, Conscientiousness, and Extraversion. Previous research has demonstrated that personality traits are linked to the onset, severity, and trajectory of various psychiatric disorders. Additionally, personality traits have been identified as robust predictors of overall psychological well-being (Cloninger et al., 1997; Davydov et al., 2010), often assessed using the 12-item version of the General Health Questionnaire (GHQ-12) (Goldberg and Williams, 1988).

Epilepsy has the potential to moderate the associations between personality traits and mental health outcomes through various mechanisms. Firstly, epilepsy can lead to alterations in mood, cognition, and behavior, thereby influencing an individual’s personality profile (Shehata and Bateh, 2009; Wilson et al., 2009; Weisberg et al., 2011; Findikli et al., 2016; Wang et al., 2018; Bonet et al., 2019; Leong et al., 2019; Rassart et al., 2020). Secondly, the coexistence of epilepsy and mental health conditions can interact and mutually influence each other, making it challenging to disentangle the specific contributions of personality traits to mental health outcomes. Moreover, stress, a common trigger for seizures, can be exacerbated by certain personality traits, further impacting mental health. Additionally, antiepileptic medications may have cognitive and emotional side effects that can influence personality expression and mental well-being. Lastly, the social stigma associated with epilepsy can contribute to psychological distress (Mula and Kaufman, 2020), with certain personality traits exacerbating the impact of societal factors on mental health outcomes. By exploring the moderating role of epilepsy, this research aims to shed light on the nuanced interplay between personality traits and mental health outcomes, providing valuable insights for tailored interventions and support for individuals with epilepsy and mental health concerns.

Although there have been studies investigating the associations between personality traits and mental health problems in the general population, much remains unknown about how personality traits relate to mental health problems specifically in individuals with epilepsy. This knowledge gap is significant considering that improving patient outcomes is one of the primary goals of healthcare professionals specializing in epilepsy. Therefore, the objective of the present study was to examine the association between personality traits and mental health in individuals with epilepsy and compare it to that of healthy controls.

## 2. Methods

### 2.1. Data

The cross-sectional study used data from Understanding Society: UK Household Longitudinal Study (UKHLS), which is a longitudinal panel study that collects data from households in the United Kingdom since 1991 (University of Essex, Institute for Social and Economic Research, 2022). The UKHLS is a large-scale panel survey of households in the United Kingdom. The survey aims to collect information on various aspects of social and economic life in the UK, including education, employment, health, and housing. The Understanding Society UKHLS uses a complex sample design to ensure that the sample is representative of the UK population. The sample was selected using a multi-stage stratified sampling design that involved selecting geographic areas (primary sampling units), households within those areas (secondary sampling units), and individuals within households. The sample was drawn from the Royal Mail’s Postal Address File, which covers all addresses in the UK, including private households, communal establishments, and businesses. The sample was stratified by region, local authority, and area deprivation. In addition, an oversample of ethnic minority groups was included to ensure that there were sufficient numbers of these groups in the sample.

All participants first completed a question regarding if they have been clinically diagnosed with epilepsy in Wave 1 (collected between 2009 and 2010). Then at each wave until Wave 3, participants were asked again if they have been newly diagnosed with epilepsy. Personality and demographic questions were asked in Wave 3 (collected between 2011 and 2012). Participants who completed all questions of interests were included with as people with any missing variables (*N* = 2,232) were removed from further analysis. Thus, there were 334 people with epilepsy with a mean age of 45.14 ± 15.88 years old (41.32% males) and 26,484 healthy controls (42.5% males) with a mean age of 48.71 ± 17.04 years old.

### 2.2. Measures

#### 2.2.1. Epilepsy

Self-reported epilepsy is a valid approach for identifying epilepsy at the population level (Brooks et al., 2012). Specifically, the sensitivity was 84.2%, specificity was 99.2%, and positive predictive value (PPV) was 73.5% for self-reported lifetime epilepsy (Brooks et al., 2012). Epilepsy was retrospectively assessed using the question, “Has a doctor or other health professional ever told you that you have any of these conditions? “Epilepsy”. During Wave 1. In subsequent waves, participants were asked if they had received a new diagnosis of epilepsy. Those who reported being clinically diagnosed with epilepsy at any point were classified as individuals with epilepsy.

#### 2.2.2. Personality traits

Personality was measured using the 15-item version of the Big Five Inventory (John et al., 1991) with a Likert scale ranging from 1 (“disagree strongly”) to 5 (“agree strongly”). Scores were reverse coded when appropriate. The exact set of questions used to ask participants can be found: https://www.understandingsociety.ac.uk/documentation/mainstage/dataset-documentation/term/personality-traits?search_api_views_fulltext=. Internal consistency was evaluated using Cronbach’s alpha, and the values for each trait were: Neuroticism = 0.69, Openness = 0.66, Extraversion = 0.60, Agreeableness = 0.57, and Conscientiousness = 0.54. Previous studies have also demonstrated the reliability of this short questionnaire through test–retest correlations, as well as its convergent and discriminant validity (Hahn et al., 2012; Soto and John, 2017).

#### 2.2.3. Mental health

GHQ-12 was used to measure general (non-psychiatric) mental health, which is a 12-item unidimensional inventory (Mula and Kaufman, 2020). The GHQ-12 used the Likert method of scoring ranges from 0 (“Not at all”) to 3 (“Much more than usual”). A summary score across all the 12 items was used to represent mental health. A higher score means worse mental health. Cronbach’s alpha for this inventory is 0.78. The GHQ-12 is characterized with good validity and reliability (Hankins, 2008).

#### 2.2.4. Control variables

Control variables included age, sex, monthly income, the highest educational qualification, present marital status, and the number of close friends. Specifically, age, monthly income, and the number of close friends were coded as what they were (continuous), sex was coded as male (1) vs. female (2), and the highest educational qualification was coded as below college (1) vs. college (2), marital status was coded as (1) single vs. (2) married.

### 2.3. Analysis

A hierarchical regression was conducted to examine whether epilepsy moderates the associations between personality traits and mental health. Epilepsy status, control variables, personality traits, and interactions between personality traits and epilepsy status were included as predictors to predict mental health as measured by the GHQ-12. Participants were divided into two groups: individuals with epilepsy and those without epilepsy. Two separate multiple regressions were performed to analyze the associations between personality traits and mental health in each group. Control variables, including age, sex, monthly income, highest educational qualification, present marital status, and the number of friends, as well as personality traits (Neuroticism, Agreeableness, Openness, Conscientiousness, and Extraversion), were included as predictors in the regression models. The GHQ-12 summary score was used as the dependent variable for individuals with and without epilepsy, respectively. All analyses were conducted using MATLAB 2018a.

## 3. Results

Descriptive statistics were illustrated in Table 1. The current study found that epilepsy status significantly moderates the association between Neuroticism and mental health (*b* = 0.49, *p* < 0.01, 95% C.I. [0.14, 0.85]; Table 2). Specifically, control variables and personality traits explained 24% (*R*^2 = 0.24) total variances of mental health in healthy controls, with Neuroticism positively related to worse mental health (*b* = 1.73, *p* < 0.001, 95% C.I. [1.70, 1.78]), Openness (*b* = −0.06, *p* < 0.05, 95% C.I. [1.70, 1.78]), Conscientiousness (*b* = −0.42, *p* < 0.001, 95% C.I. [1.70, 1.78]), and Extraversion (*b* = −0.16, *p* < 0.001, 95% C.I. [1.70, 1.78]) negatively related to worse mental health. However, the predictors explain 31% (*R*^2 = 0.31) total variances of mental health in people who have been clinically diagnosed with epilepsy with Neuroticism (*b* = 2.21, *p* < 0.001, 95% C.I. [1.76, 2.66]) positively related to worse mental health and Conscientiousness (*b* = −0.61, *p* < 0.05, 95% C.I. [−1.21, −0.01]) negatively related to worse mental health. The full regression results can be found in Table 3.

**Table 1:** Descriptive statistics for healthy control and people with epilepsy.

	Healthy controls		People with epilepsy	
	Mean	S.D.	Mean	S.D.
Age	48.71	17.04	45.14	15.88
Monthly income	1436.59	1337.06	1248.14	1067.87
Number of close friends	5.19	5.23	5.45	5.78
GHQ-12	11.05	5.45	12.67	6.75
Neuroticism	3.54	1.45	3.91	1.57
Agreeableness	5.66	1.04	5.64	1.13
Openness	4.54	1.32	4.34	1.40
Conscientiousness	5.51	1.11	5.29	1.17
Extraversion	4.58	1.31	4.60	1.30
	*N*	%	*N*	%
Sex				
Male	11,257	42.50	138	41.32
Female	15,227	57.50	196	58.68
Highest educational qualification				
Below college	17,981	67.89	258	77.25
College	8,503	32.11	76	22.75
Legal marital status				
Single	11,904	44.95	165	49.40
Married	14,580	55.05	169	50.60

**Table 2:** The estimates (*b*) of the hierarchical regression model by taking demographics, the number of close friends, epilepsy status, personality traits, and epilepsy status by personality traits interactions as the predictors and GHQ-12 summary score as the predicted variable.

	*b*
Epilepsy status	−2.04***
Age	0.01
Sex	0.17***
Monthly income	0.00**
Highest educational qualification	−0.25*
Legal marital status	−0.58***
Number of close friends	−0.03***
Neuroticism	1.70***
Agreeableness	−0.02
Openness	−0.04
Conscientiousness	−0.41***
Extraversion	−0.16***
Epilepsy status * Neuroticism	0.49**
Epilepsy status * Agreeableness	0.17
Epilepsy status * Openness	0.12
Epilepsy status * Conscientiousness	−0.07
Epilepsy status * Extraversion	−0.03
*R*^2	0.25

All numbers are rounded up to two digits. *p < 0.05, **p < 0.01, and ***p < 0.001.

**Table 3:** The estimates (*b*) of multiple regression models for healthy controls and people who have been diagnosed with epilepsy by taking demographics, the number of close friends, and personality traits as the predictors and GHQ-12 summary score as the predicted variable.

	Healthy controls	People with epilepsy
Age	0.01***	0.01*
Sex	0.18**	0.38
Monthly income	0.00*	0.00
Highest educational qualification	−0.25***	−0.56
Legal marital status	−0.58***	−0.03
Number of close friends	−0.03***	0.05
Neuroticism	1.73***	2.21***
Agreeableness	−0.03	0.12
Openness	−0.06*	0.15
Conscientiousness	−0.42***	−0.61*
Extraversion	−0.16***	−0.34
*R*^2	0.24	0.31

All numbers are rounded up to two digits. *p < 0.05, **p < 0.01, and ***p < 0.001.

## 4. Discussion

The aim of the current study was to examine the associations between personality traits and mental health in people with epilepsy and compare them with those of healthy controls. Using a hierarchical regression and two multiple regressions, the study analyzed data from 334 people with epilepsy (mean age = 45.14 ± 15.88 years old, 41.32% males) and 26,484 healthy controls (mean age = 48.71 ± 17.04 years old, 42.5% males). The findings revealed that epilepsy moderates the associations between Neuroticism and mental health, showing a stronger positive association with worse mental health in people with epilepsy compared to healthy controls. Additionally, Conscientiousness was negatively related to worse mental health in both people with epilepsy and healthy controls. However, it was found that Openness and Extraversion were negatively related to worse mental health in healthy controls but not in people with epilepsy.

The finding that Neuroticism was positively related to worse mental health is consistent with the literature. Indeed, individuals who score high in Neuroticism tend to experience negative effects such as anxiety, anger, self-consciousness, irritability, and fear. They also respond poorly to stressors (McCabe and Fleeson, 2012), which predisposes them to psychological distress (Barrick et al., 2001) and impulsive behavior (Costa and McCrae, 1992). Furthermore, high Neuroticism is associated with lower subjective well-being (Mitchell et al., 2021) and a higher prevalence of depression, anxiety, mood disorders, and substance abuse (Diener et al., 1999). This relationship was observed to be stronger in people with epilepsy compared to those without epilepsy, possibly due to the fact that the presence of epilepsy may exacerbate the impact on mental health.

The result that Conscientiousness was negatively related to worse mental health in both people with epilepsy and healthy controls aligns with the idea that individuals scoring high in Conscientiousness are protected from the adverse effects of mental health problems such as anxiety, depression, and perceived stress (Kotov et al., 2010). Additionally, conscientious individuals tend to possess better emotional regulation skills (Rettew et al., 2021), which, in turn, contribute to better mental health outcomes (Friedman and Kern, 2014).

Openness and Extraversion were negatively related to worse mental health in healthy controls, consistent with previous studies (Berking and Wupperman, 2012). Individuals with high Extraversion scores typically engage in more social activities, maintain stronger social connections, and experience more positive emotions. Additionally, individuals with high Openness tend to appreciate art and beauty, possess diverse interests, and seek novelty over routine, which can contribute to enhanced mental well-being. However, these associations were not observed in people with epilepsy, possibly due to the limitations imposed by the condition, which restricts their participation in various activities, thereby hindering the mental health benefits associated with Extraversion and openness. Furthermore, the larger sample size of healthy controls provided greater statistical power to detect small effects.

Despite its strengths, the current study has several limitations. Firstly, the study design is cross-sectional, making it challenging to establish causal relationships between personality traits and mental health outcomes. Secondly, the study did not account for the current state of epilepsy, potentially overlooking participants who may have recovered from the condition. Thirdly, the study did not assess whether individuals with epilepsy were experiencing active seizures, which could influence the observed results.

## 5. Conclusion

Taken together, the current assessed how personality traits are associated with mental health in people with epilepsy and healthy controls with this relationship stronger in people with epilepsy. The current study found that Neuroticism is positively related to worse mental health in both people with and without epilepsy, but Conscientiousness was negatively related to worse mental health in both people with and without epilepsy. Moreover, Openness and Extraversion were negatively related to worse mental health in healthy controls but not in people with epilepsy.

## 6. Implications

Clinicians should use findings from this study to detect people with epilepsy who may be at high risk of poor mental health based on their personality traits. Specifically, attention must be drawn to individuals who score high in Neuroticism but low in Conscientiousness to prevent them from suffering from mental health issues.

## Data availability statement

Publicly available datasets were analyzed in this study. This data can be found here: https://www.understandingsociety.ac.uk.

## Ethics statement

The studies involving human participants were reviewed and approved by University of Essex. Written informed consent to participate in this study was provided by the participants’ legal guardian/next of kin.

## Author contributions

The author confirms being the sole contributor of this work and has approved it for publication.

## Funding

This work was supported by the Imperial Open Access Fund.

## Conflict of interest

The author declares that the research was conducted in the absence of any commercial or financial relationships that could be construed as a potential conflict of interest.

## Publisher’s note

All claims expressed in this article are solely those of the authors and do not necessarily represent those of their affiliated organizations, or those of the publisher, the editors and the reviewers. Any product that may be evaluated in this article, or claim that may be made by its manufacturer, is not guaranteed or endorsed by the publisher.
